# Influence of continuous renal replacement therapy on the plasma concentration of tigecycline in patients with septic shock: A prospective observational study

**DOI:** 10.3389/fphar.2023.1118788

**Published:** 2023-03-09

**Authors:** Fang Huang, Wen-Xiang Cao, Yu-Ying Yan, Tian-Tian Mao, Xian-Wen Wang, Dan Huang, Yu-Shuang Qiu, Wen-Jie Lu, Dong-Jie Li, Yu-Gang Zhuang

**Affiliations:** ^1^ Department of Pharmacy, Shanghai Tenth People’s Hospital, Tongji University School of Medicine, Shanghai, China; ^2^ School of Pharmacy, Nanjing Medical University, Nanjing, China; ^3^ Department of Emergency Medicine, Shanghai Tenth People’s Hospital, Tongji University School of Medicine, Shanghai, China

**Keywords:** high-dose tigecycline, continuous renal replacement therapy, septic shock, plasma concentration, dose adjustment

## Abstract

**Objective:** The influence of continuous renal replacement therapy (CRRT) on the steady-state plasma concentration of high-dose tigecycline was investigated in septic shock patients to provide references for drug dosing.

**Methods:** In this prospective observational study, 17 septic shock patients presenting with severe infections needing a broad-spectrum antibiotic therapy with high-dose tigecycline (100 mg per 12 h) in the intensive care unit were included and divided into CRRT group (*n* = 6) or non-CRRT group (*n* = 11). The blood samples were collected and plasma drug concentration was determined by SHIMADZU LC-20A and SHIMADZU LCMS 8040. The steady-state plasma concentration was compared between groups using unpaired *t*-test. Furthermore, between-groups comparisons adjusted for baseline value was also done using multivariate linear regression model.

**Results:** Peak concentration (C_max_) of tigecycline was increased in CRRT group compared to non-CRRT group, but there were no statistical differences (505.11 ± 143.84 vs*.* 406.29 ± 108.00 ng/mL, *p*-value: 0.129). Trough concentration (C_min_) of tigecycline was significantly higher in CRRT group than in non-CRRT group, with statistical differences (287.92 ± 41.91 vs*.* 174.79 ± 33.15 ng/mL, *p*-value: 0.000, adjusted *p*-value: 0.000). In safety, C_min_ was reported to be a useful predictor of hepatotoxicity with a cut-off of 474.8 ng/mL. In our studies, C_min_ of all patients in CRRT group was lower than 474.8 ng/mL.

**Conclusion:** The plasma concentration of tigecycline was increased in septic shock patients with CRRT treatment and only C_min_ shown statistical differences. No dose adjustment seems needed in the view of hepatotoxicity.

**Clinical Trial Registration:** https://www.chictr.org.cn/, identifier ChiCTR2000037475.

## Introduction

Tigecycline, a novel tetracycline, has been approved by the Chinese Food and Drug Administration for the treatment of complicated intraabdominal infection, complicated skin and skin structure infections, and adult community-acquired pneumonia since 2005 (Pankey, 2005; Zhou et al., 2022). By inhibiting the protein synthesis of bacteria by binding to the 30 S ribosomal subunit and blocking the entry of aminoacyl tRNA into the A side of the ribosome, tigecycline exhibits broad-spectrum antibacterial activity against G-positive bacteria, G-negative bacteria, and anaerobic bacteria, especially multidrug-resistant bacteria, making it an important antibiotic for critically infected patients (Qu et al., 2009; Hawser et al., 2010; Dowzicky and Chmelařová, 2011; Hu et al., 2012; Gilbert et al., 2020).

Septic shock is a major cause of death in intensive care units (ICUs), with a mortality rate of 15%–30% or even higher (Singer et al., 2016; Howell and Davis, 2017; de Grooth et al., 2018). Sepsis leads to approximately 50% of acute kidney injury (AKI) cases; therefore, septic shock patients often require continuous renal replacement therapy (CRRT), which replaces the kidney’s function of blood filtration and affects the metabolism of drugs, including anti-infective agents (Bellomo et al., 2017; Li et al., 2020). Antibiotics are the core of sepsis therapy (Marshall, 2014). Tigecycline is one of the limited options for treatment of septic shock, and high-dose tigecycline (100 mg every 12 h) is widely used in clinical practice (De Pascale et al., 2014; Geng et al., 2018; Alsemari et al., 2020; Zhao et al., 2020). Consequently, septic shock patients who undergo CRRT often require antibiotic therapy with high-dose tigecycline. Providing appropriate antibiotic exposure is extremely pivotal for these patients to improve clinical outcomes, but the influence of CRRT on the plasma concentration of high-dose tigecycline remains unclear, making drug dosing more challenging in such settings (MacArthur et al., 2004; Roggeveen et al., 2022). Broeker et al. proposed that no dose adjustment of tigecycline was necessary in critically ill patients undergoing CRRT, but Patrick M. Honoure et al. pointed out that the view was somewhat premature (Broeker et al., 2018; Honore et al., 2020).

In the present study, the influence of CRRT on the steady-state plasma concentration of high-dose tigecycline was investigated in septic shock patients to provide references for drug dosing.

## Materials and methods

### Setting and study population

A prospective observational study was performed in the 23-bed ICU of the 10th People’s Hospital of Tongji University between 2020 and 2021. The protocol was approved by the hospital’s Ethical Committee (approval number 2020KT62) and has also been registered on chictr.org.cn (ChiCTR2000037475). This study was performed in accordance with the Helsinki Declaration and its later amendments. Written informed consent was obtained from the patients or their legally authorized representatives. ICU patients meeting the criteria of septic shock according to the sepsis-3 criteria were eligible for inclusion (Singer et al., 2016). The major exclusion criteria were an age <18 years, severe hepatic failure (Child–Pugh C), hyperbilirubinemia (bilirubin level higher than 3 mg/dL), or a history of allergy to tigecycline. Seventeen patients were included and completed this study. Patients were divided into two groups according to continuous renal replacement therapy (CRRT) conditions: a non-CRRT group for patients who did not require CRRT and a CRRT group for patients who underwent CRRT at least once. The criteria for CRRT included both metabolic emergencies and persistent acute kidney injury.

### CRRT

Patients were treated with continuous veno-venous haemodiafiltration (CVVHDF) using the Baxter system equipped with AN69-ST membrane (Baxter Healthcare, Chicago, United States). The bicarbonate replacement solution was used for both dialysis at a rate of 2000 mL/h and postfilter fluid replacement (postdilution) at the same rate. The blood flow rate was 160 mL/min. Additionally, the fluid clearance was 200 mL/h. Anticoagulation was achieved with unfractionated heparin, targeting an activated clotting time 1.5-times greater than that at baseline.

### Data collection

The demographic and outcome data were collected by doctors and pharmacists, including age, gender, site of infection, comorbidities, clinical outcomes, the Acute Physiology and Chronic Health Evaluation II (APACHE II) score and the sequential organ failure assessment (SOFA) score. Laboratory parameters such as alanine transaminase (ALT), aspartate aminotransferase (AST), total bilirubin (TB), conjugated bilirubin (CB), serum albumin (ALB), pre-BNP, creatinine (Cr), and urea nitrogen (BUN) were also collected.

### Study design and sample collection

Patients were treated with 100 mg of tigecycline every 12 h intravenously. From each patient, at least two blood samples were taken after at least six doses when the serum tigecycline reached steady state: blood sample A (C_max_) was collected at 0.5 h after the completion of an intravenous infusion of tigecycline for the determination of the peak concentration, and blood sample B (C_min_) was a predose concentration taken at the end of a dosing interval (within 0.5 h before the next dose) (Xu et al., 2019). Blood samples were collected using anticoagulation tubes, centrifuged at 1,500 rpm for 10 min, and stored at −80°C until assayed.

### Drug analysis

Serum tigecycline samples were analyzed by a validated liquid chromatography with mass spectrometry (LC–MS-MS) method using SHIMADZU LC-20A and SHIMADZU LCMS 8040 Triple Quadruple MS/MS (Shimane Shimadzu Corporation, Japan). The mobile phase consisted of 0.1% formic acid water (A)—0.1% formic acid acetonitrile (B). The elution gradient was as follows: 0–1.5 min 5% B; 1.5–5 min 5%–95% B; 5–5.1 min 95%–5% B; 5.1–8 min 5% B. Tigecycline and the internal standard were eluted from an XSelect@HSS T3 column with a total run time of 8 min. Detection was carried out using mass spectrometry. For sample preparation of serum, a 50 μL serum sample was mixed with 150 μL of internal standard working solution (internal standard concentration: 100 ng/mL), vortexed for 1 min, and centrifuged for 15 min at 13,000 rpm, 4°C. Finally, the supernatant was tested. The internal standard 2-chlorophenylalanine was purchased from Sigma‒Aldrich (St. Louis, Missouri, United States).

### Endpoint

The endpoint of the study was the steady-state tigecycline concentration in the serum, which was compared between non-CRRT and CRRT patients who experienced severe infections and needed broad-spectrum antibiotic therapy with tigecycline. The steady-state tigecycline concentration in the serum was labelled C_max_ and C_min_ for each patient.

### Statistical analysis

Data are presented as the mean ± SD, median ± interquartile range (interquartile range, IQR) or numbers (%) and were compared between the non-CRRT and CRRT groups using unpaired *t*-test, the Mann‒Whitney *U* test, or Fisher’s exact test according to the distribution and category of the variables.

Adjusted analyses for the tigecycline concentration were performed using a multivariate linear regression analysis model, considering potential cofounders that were not fully balanced at baseline. In order to meet the requirements of Event Per Variable, related variables CRRT, ALB, ALT, AST, and TB were selected by bootstrapped stepwise regression method (Henderson et al., 2016). CB was not included because TB contained CB. At the same time, Cr was also not included due to CRRT replaces the kidney’s function. Bootstrapping created 1,000 resampling randomly. Backwards stepwise variable selection was performed on each bootstrap dataset, separately for each outcome (C_min_ or C_max_). The frequency that a variable was selected by the variable selection procedure on the 1,000 bootstrap samples (bootstrap inclusion frequency, BIF) was used to identify important variables that should be further investigated. Variables with BIF ≥ 50% were included in the final multivariate linear regression model.

Statistical analyses were performed by IBM SPSS Statistics, version 19, and SAS, version 9.4. A *p*-value of <0.05 was deemed statistically significant.

## Results

### Demographic and clinical data

According to the inclusion criteria, a total of 17 septic shock patients in the ICU were enrolled in this study and were categorized into the CRRT group (*n* = 6) or non-CRRT group (*n* = 11) considering CRRT status (Table 1).

**TABLE 1:** Characteristics of enrolled patients.

Variable	Non-CRRT^a^ (*n* = 11)	CRRT^a^ (*n* = 6)	*p* ^b^
Age (yr)	70.3 ± 9.5	68.3 ± 16.2	0.129
Male, n (%)	8 (72.7)	2 (33.3)	0.162
Renal function			
Cr (μmol/L)	82.1 ± 31.2	201.3 ± 111.9	0.004
Bun (mmol/L)	10.5 ± 6.1	16.1 ± 10.4	0.177
Liver function			
ALT (U/L)	31.15 ± 23.45	59.6 ± 36.1	0.066
AST (U/L)	25.1 (20.6, 45.5)	52.8 (29.9, 82.9)	0.063
TB (μmol/L)	10.0 ± 3.4	19.9 ± 14.0	0.146
CB (μmol/L)	4.7 ± 1.2	11.7 ± 8.1	0.086
Serum albumin (g/L)	31.6 ± 3.0	30.0 ± 5.0	0.402
preBNP (pg/mL)	1,020.0 (225.5, 1,993.0)	4,437.5 (2,484.8, 9,211.5)	0.021
Severity scores upon ICU admission			
SOFA score	7 ± 3	11 ± 2	0.006
APACHE II	17 ± 6	23 ± 3	0.043
Clinical outcomes			
Improved	11 (100)	3 (50.0)	0.029
Not improved	0	3 (50.0)	
Site of infection			
Abdomen	2 (18.2)	2 (33.3)	0.584
Urinary tract	1 (9.1)	0	1
Pulmonary	8 (72.7)	3 (50.0)	0.600
Blood	3 (27.2)	2 (33.3)	1
Comorbidies			
Hypertension	9 (81.8)	3 (50.0)	0.280
Respiratory failure	5 (45.5)	3 (50.0)	1

APACHE II, acute physiology and chronic health evaluation II; SOFA, sequential organ failure assessment; ALT, alanine transaminase; AST, aspartate aminotransferase; TB, total bilirubin; CB, conjugated bilirubin; Cr, creatinine; bun, urea nitrogen.

^a^ Values are mean ± SD, median (range), or No. (%).

^b^ Fisher`s exact test was used to compare proportions; Unpaired Student’s t-test (normal distribution) or Mann-Whitney *U* test (abnormal distribution) was used to compare continuous variables.

Patients were treated with 100 mg of tigecycline every 12 h due to infections in the abdomen (*n* = 4), lungs (*n* = 11), blood (*n* = 5), and urinary tract (*n* = 1) (Table 1). The major infection type was pneumonia, and four patients had more than one infection site. The levels of preBNP (*p*-value: 0.021) and Cr (*p* = 0.004) and the SOFA (*p*-value: 0.006) and APACHE II scores (*p*-value: 0.043) were much higher in the CRRT group than in the non-CRRT group (Table 1). The clinical outcome of the non-CRRT group was better than that of the CRRT group (*p*-value: 0.029). There were no significant differences in the remaining clinical characteristics between the two groups.

### Analysis of the steady-state plasma concentration of tigecycline in septic shock patients

The serum steady-state tigecycline concentrations in each patient are shown in Table 2; Table 3. The mean serum tigecycline concentrations for each group were calculated and are presented as the mean ± SD. The peak concentration (C_max_) of tigecycline was increased in the CRRT group compared to the non-CRRT group, but there were no significant differences between the two groups (505.11 ± 143.84 vs. 406.29 ± 108.00 ng/mL, *p*-value: 0.129) (Table 2). The trough concentration (C_min_) of tigecycline was significantly higher in the CRRT group than in the non-CRRT group, with significant differences (287.92 ± 41.91 vs. 174.79 ± 33.15 ng/mL, *p*-value: 0.000) (Table 3).

**TABLE 2:** Peak concentration of tigecycline in septic shock patients.

Variable	Patient	Non-CRRT (*n* = 11)	CRRT (*n* = 6)	*p*	^a^Adjusted *p*-value
Cmax (ng/mL**)**	Patient 1	434.71	411.17	0.129	^b^–
	Patient 2	228.83	713.65		
	Patient 3	269.97	330.52		
	Patient 4	271.46	573.16		
	Patient 5	366.97	411.65		
	Patient 6	400.47	590.55		
	Patient 7	477.28	–		
	Patient 8	481.50	–		
	Patient 9	492.05	–		
	Patient 10	504.08	–		
	Patient 11	541.91	–		
	Mean ± SD	406.29 ± 108.00	505.11 ± 143.84		

C_max_, Peak concentration; CRRT, continuous renal replacement therapy.

p: Tigecycline concentration was compared between the two groups of patients using unpaired t-test.

^a^ Adjusted p-value: Tigecycline concentration was compared between the two groups of patients using multivariate linear regression analysis model, adjusted for variables retained after bootstrap selection.

^b^ Multivariate linear regression was not performed for C_max_ because there is no variable retained after bootstrap selection.

**TABLE 3:** Trough concentration of tigecycline in septic shock patients.

Variable	Patient	Non-CRRT (*n* = 11)	CRRT (*n* = 6)	*p*	^a^Adjusted *p*-value
Cmin (ng/mL)	Patient 1	150.14	230.20	0.000	0.000
	Patient 2	133.94	328.77		
	Patient 3	122.09	271.83		
	Patient 4	155.60	306.19		
	Patient 5	185.86	256.03		
	Patient 6	200.81	334.54		
	Patient 7	173.85	–		
	Patient 8	225.34	–		
	Patient 9	162.13	–		
	Patient 10	211.24	–		
	Patient 11	201.72	–		
	Mean ± SD	174.79 ± 33.15	287.92 ± 41.91		

C_min_, trough concentration; CRRT, continuous renal replacement therapy.

p: Tigecycline concentration was compared between the two groups of patients using unpaired *t*-test.

^a^ Adjusted p-value: Tigecycline concentration was compared between the two groups of patients using multivariate linear regression analysis model, adjusted for variables retained after bootstrap selection.

Adjusted analyses for the tigecycline concentration were performed using a multivariate linear regression analysis model, including variables retained after bootstrap selection. CRRT and ALT were included into the final multivariate linear regression model for C_min_. As for C_max_, there is no variable with a BIF of ≥50% after selection (Supplementary Table S1). The differences between the two groups were statistically significant in C_min_ after adjusted for the effect of cofounding variable ALT (adjusted *p*-value for C_min_: 0.000) (Table 3). Multivariate linear regression was not performed for C_max_ because there is no variable retained after bootstrap selection (Table 2).

Regarding safety, C_min_ was reported to be a useful predictor of hepatotoxicity with a cut-off of 474.8 ng/mL. In our studies, C_min_ was lower than 474.8 ng/mL for all patients in the CRRT group (Table 3).

Therefore, both C_max_ and C_min_ of tigecycline were increased in patients receiving CRRT treatment, but only C_min_ showed significant differences. The increase in C_min_ may not contribute to hepatotoxicity.

## Discussion

In the present study, the influence of CRRT on the steady-state plasma concentration of high-dose tigecycline was investigated in septic shock patients. Seventeen patients were divided into the CRRT or non-CRRT group and received high-dose tigecycline at 100 mg every 12 h intravenously. Both C_max_ and C_min_ were increased in the CRRT group, but only C_min_ had significant differences. In multivariate regression, CRRT significantly contributed to the increase in C_min_ after adjustment for ALT. Regarding safety, all the C_min_ values in the CRRT group were lower than 474.8 ng/mL, which was reported to be a cut-off of hepatotoxicity related to C_min_. No dose adjustment seems necessary in such settings.

Septic shock is one of the most common critical illnesses in the ICU, with a mortality rate of 15%–30% or even higher (Singer et al., 2016; Howell and Davis, 2017; de Grooth et al., 2018). High-dose tigecycline is recommended and widely used in septic shock patients who often receive CRRT due to sepsis-related AKI, but the influence of CRRT on the steady-state plasma concentration of tigecycline remains unclear, making the drug dosing of tigecycline more challenging in these patients (De Pascale et al., 2014; Garnacho-Montero and Ferrándiz-Millón, 2014; Bellomo et al., 2017). The work of Zhao et al. (2020) proposed that there were no significant differences in the plasma concentration of tigecycline between the CRRT and non-CRRT groups; therefore, CRRT might have little influence on tigecycline metabolism. However, in our research, an increase in the steady-state plasma concentration of tigecycline was observed in septic shock patients, and significant differences were observed in the trough concentration (Table 2). The following reasons might have contributed to the elevated tigecycline plasma concentration: first, in the patients with severe renal impairment, tigecycline clearance was decreased, and the area under the concentration-time curve was increased. Dialysis could not efficiently remove tigecycline (Korth-Bradley et al., 2012). Second, the duration of CRRT did not exceed 11 h for most patients in our study, which further decreased dialysis clearance (Broeker et al., 2018).

CRRT is one of the key issues in our research. Compared with the work of Broeker et al. (2018), there are some differences in CRRT. First, Broeker et al. selected the Ultraflux AV 1,000 S polysulfone membrane, while we chose the AN69-ST membrane (Broeker et al., 2018). Compared with the polysulfone membrane, the AN69-ST membrane absorbed more tigecycline (Onichimowski et al., 2020). However, there were still tigecycline adsorption losses inside the Ultraflux AV 1000 S polysulfone membrane (Broeker et al., 2018). Second, only the CVVHDF modality was applied in our research, while both CVVHDF and continuous veno-venous haemodialysis (CVVHD) were chosen by Broeker et al. (2018). Tigecycline clearance during CVVHDF (2.71 L/h) was more efficient compared to CVVHD (1.69 L/h) (Honore et al., 2020). Third, tigecycline is highly dialytic. The duration of CRRT did not exceed 11 h for most patients in our study, while it reached 24 h in the work of Broeker et al. (2018), which largely reduced dialysis clearance in our research and may account for our results.

In our research, C_max_ of tigecycline was elevated in the CRRT group, but there were no significant differences between the groups. C_min_ of tigecycline was significantly increased in the CRRT group (Table 2). An increased plasma concentration might lead to toxicity. Previous studies suggested that high-dose tigecycline was safe for critically ill patients (De Pascale et al., 2014; Zha et al., 2020). Hepatotoxicity is a serious adverse reaction related to tigecycline. C_min_ was reported to be a useful predictor of hepatotoxicity with a cut-off of 474.8 ng/mL (Fan et al., 2020). In our studies, C_min_ of all patients in CRRT group was lower than 474.8 ng/mL, which means the increase of C_min_ did not contribute to hepatotoxicity in our research. Therefore, we proposed that no dose adjustment seems necessary in such settings. We would be much more cautious to recommend therapeutic drug monitoring of tigecycline for patients undergoing CRRT considering individual variations (Zhao et al., 2020).

Despite the interesting findings in our research, some limitations exist. First, in our research, we mainly focused on the impact of CRRT with the most widely used unfractionated heparin anticoagulation on septic shock patients, and partially compensated for the lack of population pharmacokinetic data for these patients (Brandenburger et al., 2017; Frías et al., 2022). However, citric acid anticoagulants have been increasingly used in clinical practice. Since the protein binding of tigecycline is affected by divalent cations such as calcium, citric acid anticoagulants may theoretically affect the concentration of tigecycline (Dorn et al., 2018). Therefore, the potential effects of CRRT with citrate anticoagulation on the tigecycline concentration should be further investigated in future studies. Second, in the present study, the correlation between hypoalbuminemia and high-dose tigecycline was not studied due to low incidence, which should be further investigated in large clinical trials in the future. Third, our sample size was relatively small, resulting in insufficiently robust results. However, we still showed them in this article since the results were interpretable. Larger studies are needed in the future. Despite the limitations, our research partially compensates for the lack of population pharmacokinetic data in a vulnerable population and provides references for drug dosing.

In conclusion, CRRT increased the steady-state plasma concentration of tigecycline in septic shock patients who received a high dosage at 100 mg per 12 h. Only C_min_ showed significant differences, and the increased C_min_ in CRRT patients may not contribute to hepatotoxicity. Therefore, no dose adjustment seems needed in view of hepatotoxicity. Therapeutic drug monitoring of tigecycline is recommended in such settings.

## Data Availability

The raw data supporting the conclusion of this article will be made available by the authors, without undue reservation.
