# Supplementary material for: Influence of continuous renal replacement therapy on the plasma concentration of tigecycline in patients with septic shock: A prospective observational study
Source: Front Pharmacol. 2023 Mar 9;14:1118788. doi: 10.3389/fphar.2023.1118788 (PMC10034132; doi:10.3389/fphar.2023.1118788)
Supplement: Supplementary file 1 [file Table1.pdf]

Supplemental data

Table S1. BIF of variables (variable selection for serum concentration)

Variables	C_{min}	C_{max}
CRRT	99.5%*	32.5%
ALT	50.1%*	29.7%
AST	35.4%	26.3%
TB	20.2%	16.6%
ALB	21.1%	31.7%

[*]Selected in final multivariate models. Variables were selected by bootstrapped stepwise regression method.

BIF: bootstrap inclusion frequency.
